# Factors Affecting Painkillers, Sedatives/Hypnotics, Nicotine, and Unhealthy Alcohol Use Among Gay and Bisexual Men in Taiwan

**DOI:** 10.3390/ijerph17030851

**Published:** 2020-01-29

**Authors:** Dian-Jeng Li, Shiou-Lan Chen, Yu-Ping Chang, Cheng-Fang Yen

**Affiliations:** 1 Graduate Institute of Medicine, College of Medicine, Kaohsiung Medical University, Kaohsiung 80708, Taiwan; 2 Department of Addiction Science, Kaohsiung Municipal Kai-Syuan Psychiatric Hospital, Kaohsiung 80276, Taiwan; 3 School of Nursing, The State University of New York, University at Buffalo, New York, NY14214–3079, USA; yc73@buffalo.edu; 4 Department of Psychiatry, Kaohsiung Medical University Hospital, Kaohsiung 80708, Taiwan

**Keywords:** sexual minority, prescription drug, smoking, alcohol, homophobic bullying

## Abstract

Substance use has become a major health problem globally for sexual minorities. However, few studies have explored multi-dimensional factors associated with smoking, drinking, and prescription drug use. We aimed to investigate the factors affecting painkiller, sedative/hypnotic, nicotine and unhealthy alcohol use among gay and bisexual men in Taiwan. We recruited 500 gay or bisexual men and assessed their experiences of using painkillers, sedatives/hypnotics, nicotine, alcohol and multi-dimensional factors with self-reported questionnaires. Multivariate logistic regression with a forward stepwise model was used to verify the factors associated with substance use. Overall, 9.4%, 5.4%, and 13.8% of the participants reported using painkillers, sedatives/hypnotics, and nicotine, respectively, and 5.6% reported unhealthy alcohol use. Victims of traditional homophobic bullying in childhood and adolescence were more likely to report nicotine use, sedative/hypnotic use, and unhealthy alcohol use in early adulthood than non-victims. Missing classes or truancy at senior high school was associated with painkiller and sedative/hypnotic use in early adulthood. Traditional homophobic bullying and missing classes or truancy in childhood and adolescence predicted substance use in early adulthood among the gay and bisexual men in this study. Timely preventions and interventions for substance use are crucial for gay and bisexual men, especially for those who experience homophobic bullying and missing classes or truancy.

## 1. Introduction

### 1.1. Substance Use in Sexual Minorities

Substance use has become a major health concern globally for sexual minorities. Problematic substance use increases the mental health burden of sexual minorities. A previous study reported that a third of young men who have sex with men (MSM; 90% of them identified themselves as gay or bisexual men) who used club drugs (e.g., MDMA or ketamine) had attempted suicide, and that more than half of MSM who regularly use club drugs had a high level of depressive symptoms [1]. Another study reported that polydrug use among gay and bisexual men is predominantly associated with HIV infection and high-risk sexual practices [2]. Although many studies have focused on substance use in sexual minorities, few have explored the use of substances other than illicit drugs, such as prescription drugs, in sexual minorities.

### 1.2. Use of Painkillers and Sedatives/Hypnotics in Sexual Minorities

There has recently been increased interest in the misuse of prescription drugs. Painkillers are the most frequently used prescription drug worldwide [3], and painkiller misuse has steadily increased in both the United States and United Kingdom in recent years [4,5]. For sexual minority men, painkillers are also the most prevalent prescription drug, and the risk of misuse cannot be neglected [6]. An epidemiological study regarding prescription drug misuse demonstrated that being bullied at school were associated with painkiller misuse [7]. However, few studies have explored the factors associated with painkiller use in sexual minorities.

Besides painkillers, the use of sedatives/hypnotics such as benzodiazepines is also an important public health concern. A previous study indicated that approximately 12.5% of US adults used benzodiazepines in 2015 [8]. Benzodiazepines activate GABA/barbiturate receptor sites to produce muscle relaxant effects [7], and they interact with α GABA_A_ subunits to enhance sedative and anxiolytic reactions [9]. The weak reinforcing effect of benzodiazepines can lead to their abuse [10]; however, few studies have explored the use of sedatives/hypnotics in sexual minorities. A cohort survey in which 90.2% of the participants were gay and bisexual men demonstrated a 10.2% rate of the self-reported use of benzodiazepines in the past 6 months [11]. Another study indicated that frequent transactional sex was associated with benzodiazepine use for MSM of whom 92.1% were gay and bisexual men [12]. Therefore, the risk factors for sedative/hypnotic use in gay and bisexual men warrant further study.

### 1.3. Smoking and Alcohol Use in Sexual Minorities

Although the prevalence of smoking has gradually fallen in the general population [13], it is still a major concern in sexual minorities. A nationwide study in the US reported that 20.6% of sexual minority individuals smoked versus 14.9% of heterosexual individuals [14]. Gay and bisexual men have also been reported to have a 1.4- to 1.6-fold higher rate of smoking than heterosexual men [15]. Several factors associated with patterns of smoking in sexual minorities have been identified. For example, sexual minority women who smoke have been reported to have fewer economic, social and psychological resources than non-smoking sexual minority women [16].

On the other hand, unhealthy alcohol use, defined as drinking to a level above the amount or frequency recommended for the general population [17], often results in higher morbidity and mortality [18], and impacts the mental health of sexual minorities. The National Health Interview Survey in the US reported that gay and bisexual men were more likely to report heavy drinking than heterosexual men [19]. Several studies have explored the impact of social factors on alcohol use specific to gay and bisexual men, and found that they prefer to gather at bars to socialize and meet new partners more than the general population [20], and that this increases their exposure to alcohol-related harm [21]. Moreover, higher income, currently smoking, and greater social discrimination have been associated with a greater likelihood of high-risk drinking in sexual minority men [13]. Further research on the associated risk factors is crucial for clinicians to be able to early identify such issues and initiate appropriate management strategies.

### 1.4. Aims of This Study

We previously comprehensively explored the factors associated with illegal substance use in early adulthood for gay and bisexual men, including school performance, homophobic bullying and social support, and gender-related issues in childhood and adolescence [22]. This research piqued our interest on the use of substances other than heroin, methamphetamines and club drugs. In Taiwan, alcoholic beverages and tobacco are restricted to those who are older than 20 years of age. People can get painkillers and sedatives/hypnotics at a pharmacy with a doctor’s prescription. The difference in the availability between “entirely illegal” drugs and “partially restricted” drugs may lead to different effects on health outcomes among gay and bisexual men. Furthermore, research on the association between multi-dimensional factors and “partially restricted” substances in gay and bisexual men is still insufficient. Given these gaps in knowledge, the aim of this study was to survey the impacts of multiple factors including traditional/cyber homophobic bullying, disclosure of sexual orientation, perceived family support, and school difficulties during childhood and adolescence on the use of painkillers, sedatives/hypnotics, nicotine and unhealthy alcohol use among sexual minority men in Taiwan.

## 2. Materials and Methods 

### 2.1. Participants

The detailed protocol of the current study has been described in our published work [22]. In brief, we recruited gay or bisexual men aged between 20 and 25 years through online and printed advertisements posted on social networking sites and lesbian, gay, bisexual, and transgender (LGBT) clubs. Participants were recruited from August 2015 to July 2017. Individuals who exhibited any cognitive impairment (e.g., intellectual disability or substance intoxication) that could hinder understanding of the purpose of the study or completing the questionnaires were excluded. Before assessment, informed consent was obtained from all participants. In total, 500 males (371 gay men and 129 bisexual men) were recruited with a mean age of 22.94 ± 1.57 years. All of them provided informed consent prior to their assessment. The study was approved by the Institutional Review Board of Kaohsiung Medical University Hospital (KMUHIRB-F(I)-20150026).

### 2.2. Measures

#### 2.2.1. Substance Use

We used the Drug Use Disorders Identification Test-Extended (DUDIT-E) to verify a history of multiple substance use [23]. It had been developed for sequential clinical assessment of drug use. The concurrent validity of D–score is reported to be acceptable, and test-retest reliability is 0.79, indicating an excellent intraclass correlation [23]. The participants were asked about the frequencies of painkiller, sedative/hypnotic, and tobacco (nicotine) use in the preceding month. Participants who had used any kind of these substances more than once in the preceding month were classified as having specific substance use. A massive epidemiological study indicated that drinking more than 3 to 4 times weekly significantly increased cardiovascular and cancer mortality in men [24]. To identify those with unhealthy alcohol use, we set the cutoff point at “drinking more than twice per week”, including the categories of “twice to thrice per week” and “four times per week or above”. Hence, this cutoff point can cover those who drink 3 to 4 times weekly. On the other hand, we did not exclude those who are taking painkillers or sedatives/hypnotics in accordance with their medically intended use due to unrecorded information from DUDIT-E.

#### 2.2.2. Experiences of Traditional and Cyber Homophobic Bullying

We used six items from the Chinese version of the self-reported School Bullying Experience Questionnaire (C-SBEQ) [25] to assess the experiences of traditional homophobic bullying according to their gender role nonconformity or sexual orientation at school, after-school classes, tutoring schools, and part-time workplaces at elementary (grades 1–6), junior high (grades 7–9), and senior high (grades 10–12) school stages. Multiple types of traditional homophobic bullying were evaluated, including social exclusion, name calling, verbal abuse, physical abuse, forced work, and confiscation of money, school supplies and snacks. The responses for these items were graded on a 4-point Likert scale. A previous study reported that the C-SBEQ had acceptable reliability and validity [25]. The Cronbach α value of the scale for evaluating traditional homophobic bullying was 0.82 in the present study. Based on the results of a previous study [25], we classified the participants who rated 2 or 3 for any item as self-reported victims of traditional homophobic bullying.

We used three items from the Cyberbullying Experiences Questionnaire [26] to evaluate the participants’ experiences of cyberbullying based on their gender role nonconformity or sexual orientation at elementary, junior high, and senior high school stages. These three items addressed the experiences of others posting mean or unpleasant comments; others posting upsetting pictures, photos, or videos; and online rumor-spreading through emails, blogs, social media platforms, and pictures or videos. The Cronbach α value of the scales for evaluating homophobic cyberbullying was 0.81 in this study. Based on the results of a previous study [25], we classified the participants who rated 1 for any item as self-reported victims of homophobic cyberbullying.

#### 2.2.3. Demographic and Family Characteristics

We recorded information of the participants’ age, educational level, parental marriage status, and parental education levels. The participants were classified into those with a high education level (college or above) and those with a low education level (high school or below). They were also classified into those with high parental education levels (both parents completed 9 years of compulsory fundamental education) and those with low parental education levels (any parent did not complete 9 years of compulsory fundamental education).

#### 2.2.4. Sexual Orientation Characteristics

The participants’ sexual orientation (homosexual or bisexual) and time of disclosure of sexual orientation (elementary school, junior high school, senior high school, and college) was recorded.

#### 2.2.5. Social Support

The Chinese version of the 5-item self-administered Family Adaptation, Partnership, Growth, Affection, Resolve (APGAR) Index was used to measure the participants’ satisfaction with family support during their childhood and adolescence [27,28]. Each item was rated on a 4-point Likert scale, with scores ranging from 0 (never) to 3 (always). In addition, we used the Peer APGAR Index, which is adapted from the Family APGAR Index, to measure the participants’ satisfaction with peer support during childhood and adolescence [29]. Higher total scores on the Family and Peer APGAR Indices indicated higher levels of family and peer support, respectively. The Cronbach α values for the Family and Peer APGAR Indices in this study were 0.86 and 0.87, respectively.

#### 2.2.6. School Characteristics

The participants were asked to rate their subjective satisfaction with their academic performance at elementary, junior high, and senior high schools using a 4-point Likert scale, ranging from 0 (very satisfied) to 3 (not satisfied at all). The participants who responded 2 or 3 were identified as being dissatisfied with their academic performance. The tendency to miss classes or be truant at elementary, junior high, and senior high schools was evaluated using a 4-point Likert scale, ranging from 0 (never) to 3 (very frequent). The participants who did not answer 0 on any item were classified as having a tendency to miss classes or be truant.

### 2.3. Procedure

This cross-sectional study used a paper-and-pencil questionnaire. Research assistants explained the procedures and methods for completing the questionnaires to the participants individually. The participants could ask any question if they had difficulty in completing the questionnaires, and the research assistants resolved any problems.

### 2.4. Statistical Analysis

Initially, the demographic, sexual orientation, family, school and social support characteristics were summarized. Univariate logistic regression was used to examine potential factors associated with painkillers, sedatives/hypnotics, nicotine, and unhealthy alcohol use. All significant variables identified in the first step were then entered into a forward stepwise logistic regression model to determine the best predictors. Prevalence ratios (PRs) and 95% confidence intervals (CIs) were used to present the statistical significance. To estimate the correlation or dependency between variables, the Pearson correlation was used to determine the linear relationships between bullying (traditional or cyber) and missing classes/truancy or satisfaction with academic performance. All tests were 2-tailed, and statistical significance was set at *p* < 0.05. All data were processed using SPSS version 23.0 for Windows (SPSS Inc., Chicago, IL, USA).

## 3. Results

### 3.1. Patient Variables

Table 1 presents the distributions and frequencies of substances use in the 500 participants. In total, 9.4%, 5.4%, and 13.8% of the participants reported using painkillers, sedatives/hypnotics, and smoking at least once per month but less than twice per month, respectively, and 5.6% reported using alcohol at least twice per week (unhealthy alcohol use).

Data on the demographics, sexual orientation, traditional and cyber homophobic bullying, family and school characteristics, and social support of the participants are listed in Table 2, Table 3, Table 4 and Table 5. 

### 3.2. Predictors of Painkillers, Sedatives/Hypnotics, Nicotine, and Unhealthy Alcohol Use

The results of the univariate logistic regression analysis were as follows. Being victims of homophobic cyberbullying, disclosure of sexual orientation at junior high school and at senior high school, and missing classes or truancy at senior high school were significantly associated with the use of painkillers. Being victims of traditional homophobic bullying, being victims of homophobic cyberbullying, disclosure of sexual orientation at junior high school, lower Family APGAR Index score, lower satisfaction with academic performance at senior high school, missing classes or truancy at junior high school and senior high school were significantly associated with the use of sedatives/hypnotics. Lower educational level, being victims of traditional homophobic bullying, being victims of homophobic cyberbullying, lower Family APGAR Index score, higher paternal educational level (higher as indicator: PR = 2.17, *p* = 0.038; lower as indicator: PR = 0.46, *p* = 0.038), and missing classes or truancy at senior high school were significantly associated with nicotine use. Being victims of traditional homophobic bullying and being victims of homophobic cyberbullying were significantly associated with unhealthy alcohol use (Table 2, Table 3, Table 4 and Table 5).

The results of the forward stepwise logistic regression analysis were as follows (Table 6). Disclosure of sexual orientation at senior high school and missing classes or truancy at senior high school were significantly associated with the use of painkillers. Being victims of traditional homophobic bullying, lower Family APGAR Index score, and missing classes or truancy at senior high school were significantly associated with the use of sedatives/hypnotics. Being victims of traditional homophobic bullying, being victims of homophobic cyberbullying, lower educational level, higher paternal educational level (higher as indicator: PR = 2.49, *p* = 0.019; lower as indicator: PR = 0.40; *p* = 0.019), and missing classes or truancy at senior high school were significantly associated with nicotine use. Being victims of traditional homophobic bullying was significantly associated with unhealthy alcohol use.

The correlations between bullying and school factors (missing classes/truancy and satisfaction with academic performance) were estimated. The correlations with cyberbullying are as follows: truancy in elementary school (r = 0.03; *p* = 0.458), truancy in junior high school (r = 0.19; *p* <0.001), truancy in senior high school (r = 0.09; *p* = 0.037), satisfaction in elementary school (r = 0.05; *p* = 0.235), satisfaction in junior high school (r = 0.16; *p* < 0.001), satisfaction in senior high school (r = 0.07; *p* = 0.106). On the other hand, the correlation with traditional bullying are as follows: truancy in elementary school (r = 0.03; *p* = 0.459), truancy in junior high school (r = 0.11; *p* = 0.012), truancy in senior high school (r = 0.05; *p* = 0.268), satisfaction in elementary school (r = 0.12; *p* = 0.008), satisfaction in junior high school (r = 0.17; *p* < 0.001), satisfaction in senior high school (r = 0.03; *p* = 0.472). Among them, the effect sizes of significant correlations are low regarding the cutoff values of Pearson correlation.

## 4. Discussion

### 4.1. The Main Findings of the Present Study

We found that painkiller use was associated with the disclosure of sexual orientation at junior high school and missing classes or truancy at senior high school among the Taiwanese gay and bisexual men in this study. The use of sedatives/hypnotics was associated with traditional homophobic bullying in childhood and adolescence, lower family support, and missing classes or truancy at senior high school. Nicotine use was associated with traditional/cyber homophobic bullying in childhood and adolescence, higher paternal educational level, lower educational level, and missing classes or truancy at senior high school. Unhealthy alcohol use was associated with traditional homophobic bullying in childhood and adolescence.

### 4.2. Characteristics of Substance Use in Sexual Minorities

In this study, 9.4% of the recruited gay and bisexual men took painkillers at least once per month, which is different to previous studies. An epidemiological investigation in the elderly found that analgesics were the most common over-the-counter medication in 2003–2004, with a prevalence of 8.8% [3]. A study on MSM of whom 98.5% were gay and bisexual reported that 11.3% of the participants had taken painkillers in the past 3 months [30], which is comparable to our results. However, they reported a higher 3-month prevalence of sedatives (8.3%) and sleep aids (16.3%) than in our study (5.4% at least once per month). On the other hand, the smoking rate in our study (13.8% at least once per month) was lower than in a prospective study reported by Fallin et al. (25.9% for gay and 33.7% for bisexual men) [31]. The differences between the results may be due to differences in measurements. We chose “at least once per month” as the cutoff point to identify those who took painkillers continuously, and also for the use of nicotine and sedatives/hypnotics. Moreover, no previous study has investigated unhealthy alcohol use (more than 14 drinks per week) in sexual minorities.

### 4.3. Traditional/Cyber Homophobic Bullying and Disclosure of Sexual Orientation Associated with Substance Use

Traditional homophobic bullying was a major risk factor for sedatives/hypnotics, nicotine, and unhealthy alcohol use in this study. We also found an association between traditional homophobic bullying and illegal substance use in our previous work [22]. Homophobic bullying has been reported to be a serious risk factor for alcohol use among sexual minority youth [32]. In another longitudinal study, Tucker et al. did not find an association between homophobic name-calling and alcohol use within sexual minority students, although the psychological stress was significantly increased [33]. This difference may be due to the 1-year observation period in Tucker et al.’s study, and because we investigated the impact of homophobic bullying in childhood and adolescence, which may further explain the effect of alcohol use in early adulthood.

In our previous work, we found that victims of traditional homophobic bullying had more severe physical pain in adulthood [29]. Therefore, it is reasonable that the use of painkillers is associated with traditional homophobic bullying. In addition, perceived discrimination was associated with an increased risk of current smoking in a multiethnic population-based study [34], and we found a similar association in gay and bisexual men. Furthermore, we identified the impact of traditional homophobic bullying on sedative/hypnotic use, which has rarely been reported. Although we did not find that homophobic bullying was a powerful predictor for painkiller use in forward stepwise logistic regression, we found that disclosure of sexual orientation at senior high school remained a major risk factor. Disclosure of one’s sexual orientation has been reported to increase psychosocial stress in sexual minorities [35]. It is possible that individuals who disclose their sexual orientation may suffer from homophobic victimization and, consequently, substance use.

Besides traditional homophobic bullying, we also found that homophobic cyberbullying in childhood and adolescence was associated with smoking in early adulthood. Although both traditional bullying and cyberbullying were risk factors, cyberbullying has been reported to have different characteristics from traditional bullying, such as anonymity and individualistic activities [36]. Cyberbullying has also been associated with smoking in Italian adolescents [37]. We further confirmed this association among the gay and bisexual men in this study. Previous studies have used “Minority Stress Theory” [38] to verify the association between homophobic victimization and substance use in sexual minorities [39]. Investigations of the risk factors of substance use in sexual minorities may benefit the development of prevention and intervention programs. Our results also support the need for preventions and interventions for traditional/cyber homophobic bullying in gay and bisexual men.

### 4.4. School Factors and Education Level Associated with Substance Use

We found that missing classes or truancy at senior high school was significantly associated with multiple substance use in early adulthood, including painkillers, sedatives/hypnotics, and nicotine. A previous study also reported that truancy was associated with substance use in children and adolescents [40]. However, no previous study has explored this association among sexual minorities. In our previous work, we found an association between illegal substance use and truancy among gay and bisexual men [22], and the current study extends these findings to painkillers, sedatives/hypnotics, and nicotine. On the other hand, in the present study, we also found that a lower educational level was significantly associated with nicotine use. A lower educational level has been reported to be related with smoking in sexual minority women [41]. Another study reported that LGBT individuals with a higher education level had lower odds of smoking [42]. Our study showed similar results for nicotine use among gay and bisexual men.

### 4.5. Family Factors Associated with Substance Use

The use of sedatives/hypnotics and nicotine was associated with a lower Family APGAR Index score in this study, which means poorer family support. Poor family management among adolescents has also been associated with increased alcohol and other drug use [43]. In addition, a previous study indicated that family rejection predicted substance use and related problems in sexual minorities [44]. A more supportive LGBT environment in schools and communities has been reported to result in less substance use in sexual minority adolescents [45]. We also identified the importance of family support in childhood and adolescence to prevent substance use in early adulthood; however, we did not find a significant association between peer support during childhood and adolescence and substance use in early adulthood.

We also found that a higher paternal educational level was significantly associated with nicotine and potentially related with unhealthy alcohol use, which has rarely been reported previously. Greater paternal demandingness has been significantly associated with binge drinking in Argentinean youth [46]. To date, no published study has investigated this association. We hypothesize that fathers with a higher educational level may have higher expectations of their children, which may then lead to increased stress and, consequently, substance use. 

### 4.6. Limitations

We presented the comprehensive results of the associations between multi-dimensional factors and substance use; however, several limitations of the current study should be noted. First, we did not record the doses of the substances used, so we could not precisely estimate the harm done to the participants. Second, the sources of painkillers and sedatives/hypnotics were also unrecorded, so that it was difficult to identify whether or not they had prescriptions for the drugs. This may have led to underestimation of the potential abuse of prescription drugs. Third, given that we did not ask the age at initial substance use, we could not identify temporal relationships between substance use and related factors with age. Fourth, this self-reported study obtained data on homophobic bullying of the participants, school factors and family and peer support retrospectively. Hence, recall bias may have confounded the results. Fifth, the mean age of recruited subjects was relatively young (20~25 years old), which limited to the generalizability to other birth cohorts. Finally, we recruited only gay and bisexual men, so our findings may not be applicable to lesbian or other sexual minorities, and further studies are warranted to generalize out result to the broader LGBT community in Taiwan.

## 5. Conclusions

In this study, we found that multi-dimensional factors were associated with substance use, including painkillers, nicotine, sedatives/hypnotics, and unhealthy alcohol use. To be specific, we provided new information that homophobic bullying and school-related factors in childhood and adolescence affected substance use in early adulthood. Our results revealed that mental health professionals should regularly assess histories of homophobic bullying and school-related factors when assessing gay and bisexual men with substance use. Gay-affirmative cognitive behavioral therapy has been shown to be beneficial for victims of homophobic bullying in childhood and adolescence [47], and timely treatment for psychological trauma from bullying may prevent substance use due to maladaptive behaviors. We suggest that gender-friendly/neutral policy in school is beneficial to prevent substance use, and we provide useful information for the development of prevention and early intervention programs for substance use in gay and bisexual men.

## Figures and Tables

**Table 1:** Distribution of and frequency of painkillers, sedative/hypnotic drugs, nicotine, and unhealthy alcohol use among 500 participants.

Type of Substance	Painkillers		Sedative/Hypnotics		Nicotine		Unhealthy Alcohol Use	
Distribution	*n*	%	*n*	%	*n*	%	*n*	%
Never used in recent one year	414	82.8	456	91.2	417	83.4	178	35.6
Ever used but less than once per month	39	7.8	17	3.4	14	*2.8*	*94*	*18.8*
Once per month but less than twice per month	21	4.2	5	1.0	9	1.8	107	21.4
Twice to four times per month	18	3.6	9	1.8	7	1.4	93	18.6
Twice to thrice per week	5	1.0	4	0.8	5	1.0	*15*	*3.0*
Four times per week or above	3	0.6	9	1.8	48	9.6	13	2.6

**Table 2:** Associations of demographic factors, sexual orientation and victimization of homophobic bullying with painkillers and sedatives/hypnotics use examined by univariate logistic regression (*n* = 500).

			Painkillers			Sedatives/Hypnotics		
	Mean	SD	PR	95% CI	*p*	PR	95% CI	*p*
Age (years)	22.94	1.57	1.07	0.88–1.29	*0.525*	*1.01*	*0.78–1.29*	*0.971*
	*n*	%	OR	95.0% of CI	*p*	OR	95% CI	*p*
Education level								
High (college or above)	450	90						
Low (high school or below)	50	10	1.36	0.55–3.38	*0.508*	0.71	*0.16–3.08*	*0.646*
Sexual orientation identity								
Gay	371	74.2						
Bisexual	129	25.8	0.87	0.43–1.76	*0.694*	1.01	*0.42–2.44*	*0.988*
Victims of homophobic traditional bullying								
No	310	62						
Yes	190	38	1.80	0.99–3.30	0.055	3.50	1.54–7.96	**0.003**
Victims of homophobic cyberbullying								
No	299	59.8						
Yes	201	40.2	1.97	1.07–3.60	**0.029**	2.67	1.20–5.96	**0.016**
Time to disclose sexual orientation								
At elementary school								
No	476	95.2						
Yes	24	4.8	1.40	0.40–4.90	0.595	0.75	0.10–5.79	0.785
At junior high school								
No	365	73						
Yes	135	27	2.18	1.18–4.03	**0.013**	2.28	1.04–5.00	**0.004**
At senior high school								
No	215	43						
Yes	285	57	2.37	1.20–4.68	**0.013**	0.94	0.43–2.05	0.940
At college or above								
No	53	10.6						
Yes	447	89.4	5.97	0.81–44.17	0.08	1.51	0.35–6.57	0.582

CI = Confidence interval; PR = Prevalence ratio, ratio of odds of illegal substance use vs. non-use among participants; SD = Standard deviation; **Bolds**: statistical significance (*p* < 0.05).

**Table 3:** Family, peer and school factors related to painkillers and sedatives/hypnotics use examined by univariate logistic regression (*n* = 500).

			Painkillers				Sedatives/Hypnotics	
	Mean	SD	PR	95% CI	*p*	PR	95% CI	*p*
Perceived family support on the APGAR	8.49	3.83	0.93	0.86–1.01	0.08	0.81	0.73–0.90	**<0.001**
Perceived peer support on the APGAR	11.42	2.89	0.98	0.93–1.16	0.516	0.91	0.81–1.02	0.112
	*n*	%	OR	95.0% CI	*p*	OR	95% CI	*p*
Parental marital status								
Married and living together	328	65.6	-	-	-			
Separated or divorced	136	27.2	1.44	0.74–2.81	0.286	2.17	0.98–4.82	0.057
Widowed	36	7.2	2.32	0.89–6.09	*0.086*	*0.64*	*0.08–5.02*	*0.672*
Paternal education level								
High (senior high school or above)	385	77						
Low (junior high school or below)	115	23	0.66	0.30–1.46	0.309	0.40	0.12–1.36	0.144
Maternal education level								
High (senior high school or above)	388	77.6						
Low (junior high school or below)	112	22.4	1.07	0.52–2.17	0.862	0.42	0.12–1.41	0.160
Satisfaction with academic performance								
In elementary school								
High	401	80.2						
Low	99	19.8	1.44	0.72–2.89	0.302	1.77	0.75–4.17	0.193
In junior high school								
High	336	67.2						
Low	164	32.8	1.44	0.78–2.67	0.244	1.69	0.77–3.70	0.189
In senior high school								
High	310	62.0						
Low	190	38.0	0.92	0.49–1.71	0.917	2.50	1.13–5.51	**0.023**
Missing classes or truancy								
In elementary school								
No	456	91.2						
Yes	44	8.8	1.60	0.64–4.00	0.317	2.53	0.91–7.05	0.076
In junior high school								
No	414	82.8						
Yes	86	17.2	1.76	0.87–3.54	0.116	3.07	1.36–6.97	**0.007**
In senior high school								
No	359	71.8						
Yes	141	28.2	2.47	1.34–4.55	**0.004**	4.79	2.13–10.73	**<0.001**

CI = Confidence interval; PR = Prevalence ratio; SD = Standard deviation; **Bolds**: statistical significance (*p* < 0.05).

**Table 4:** Associations of demographic factors, sexual orientation and victimization of homophobic bullying with nicotine and unhealthy alcohol use examined by univariate logistic regression (*n* = 500).

			Nicotine				Alcohol	
	Mean	SD	PR	95% CI	*p*	PR	95% CI	*p*
Age (years)	22.94	1.57	0.93	0.79–1.09	*0.374*	*0.84*	*0.66–1.07*	*0.156*
	*n*	%	OR	95.0% of CI	p	OR	95% CI	p
Education level								
High (college or above)	450	90						
Low (high school or below)	50	10	3.53	1.82–6.82	***<0.001***	*2.06*	*0.75–5.69*	*0.162*
Sexual orientation identity								
Gay	371	74.2						
Bisexual	129	25.8	1.66	0.96–2.85	*0.068*	*1.39*	*0.61–3.15*	*0.432*
Victims of homophobic traditional bullying								
No	310	62						
Yes	190	38	2.25	1.35–3.76	**0.002**	3.14	1.42–6.96	**0.005**
Victims of homophobic cyberbullying								
No	299	59.8						
Yes	201	40.2	2.31	1.38–3.88	**0.001**	2.84	1.28–6.29	**0.01**
Time to disclose sexual orientation								
At elementary school								
No	476	95.2						
Yes	24	4.8	1.27	0.42–3.82	0.677	1.57	0.35–7.06	0.554
At junior high school								
No	365	73						
Yes	135	27	0.95	0.53–1.69	0.854	1.54	0.69–3.43	0.288
At senior high school								
No	215	43						
Yes	285	57	0.98	0.59–1.63	0. 931	1.38	0.63–3.06	0.425
At college or above								
No	53	10.6						
Yes	447	89.4	0.76	0.35–1.63	0.479	0.52	0.19–1.43	0.206

CI = Confidence interval; PR = Prevalence ratio; SD = Standard deviation; **Bolds**: statistical significance (*p* < 0.05).

**Table 5:** Family, peer and school factors associated with nicotine and unhealthy alcohol use examined by univariate logistic regression (*n* = 500).

			Nicotine			Alcohol		
	Mean	SD	PR	95% CI	*p*	PR	95% CI	*p*
Perceived family support on the APGAR	8.49	3.83	0.90	0.84–0.96	**0.002**	0.96	0.87–1.06	0.457
Perceived peer support on the APGAR	11.42	2.89	1.01	0.93–1.11	0.753	0.97	0.85–1.10	0.602
	*n*	%	OR	95.0% CI	*p*	OR	95% CI	*p*
Parental marital status								
Married and living together	328	65.6	-	-	-			
Separated or divorced	136	27.2	1.28	0.72–2.26	0.397	0.962	0.94–3.49	0.077
Widowed	36	7.2	1.69	0.70–4.11	*0.247*	*<0.001*	*<0.001-<0.001*	*0.998*
Paternal education level								
High (senior high school or above)	385	77						
Low (junior high school or below)	115	23	0.46	0.22–0.96	**0.038**	0.24	0.06–1.05	0.057
Maternal education level								
High (senior high school or above)	388	77.6						
Low (junior high school or below)	112	22.4	0.62	0.31–1.23	0.169	0.25	0.06–1.08	0.064
Satisfaction with academic performance								
In elementary school								
High	401	80.2						
Low	99	19.8	1.04	0.55–1.95	0.912	0.87	0.32–2.36	0.791
In junior high school								
High	336	67.2						
Low	164	32.8	1.19	0.70–2.03	0.513	0.97	0.43–2.19	0.939
In senior high school								
High	310	62.0						
Low	190	38.0	1.06	0.63–1.78	0.835	0.53	0.22–1.26	0.151
Missing classes or truancy								
In elementary school								
No	456	91.2						
Yes	44	8.8	0.60	0.21–1.74	0.348	0.79	0.18–3.44	0.751
In junior high school								
No	414	82.8						
Yes	86	17.2	1.72	0.94–3.14	0.081	1.66	0.68–4.03	0.265
In senior high school								
No	359	71.8						
Yes	141	28.2	2.94	1.75–4.95	**<0.001**	1.99	0.92–4.33	0.081

CI = Confidence interval; PR = Prevalence ratio; SD = Standard deviation; **Bolds**: statistical significance (*p* < 0.05).

**Table 6:** Predictors of painkillers, sedatives/hypnotics, nicotine, and unhealthy alcohol use examined using forward stepwise logistical regression.

*Predictors of Painkillers Use*	PR	95% CI	*p*
Disclosure of sexual orientation at senior high school	2.35	1.18–4.67	**0.015**
Missing classes or truancy in senior high school	2.45	1.33–4.53	**0.004**
*Predictors of sedatives/hypnotics use*	OR	95% CI	*p*
Victims of homophobic traditional bullying	2.65	1.12–6.25	**0.027**
Perceived family support on the APGAR	0.85	0.76–0.95	**0.003**
Missing classes or truancy in senior high school	4.02	1.75–9.24	**0.001**
*Predictors of nicotine use*	OR	95% CI	*p*
Victims of homophobic traditional bullying	1.92	1.10–3.34	**0.022**
Victims of homophobic cyberbullying	1.78	1.02–3.10	**0.042**
Missing classes or truancy in senior high school	2.46	1.43–4.25	**0.001**
Paternal education level	0.40	0.19–0.86	**0.019**
Education level	2.73	1.34–5.55	**0.006**
*Predictors of unhealthy alcohol use*	OR	95% CI	*p*
Victims of homophobic traditional bullying	3.14	1.42–6.96	**0.005**

PR = Prevalence ratio; **Bolds**: statistical significance (*p* < 0.05).
